# Ear health and quality of life in pet rabbits of differing ear conformations: A UK survey of owner-reported signalment risk factors and effects on rabbit welfare and behaviour

**DOI:** 10.1371/journal.pone.0285372

**Published:** 2023-07-19

**Authors:** Benedict D. Chivers, Melissa R. D. Keeler, Charlotte C. Burn

**Affiliations:** Animal Welfare Science and Ethics, Royal Veterinary College, Hertfordshire, United Kingdom; University of Wisconsin-La Crosse, UNITED STATES

## Abstract

The impacts of ear disease on animal welfare and behaviour are little documented. Ear disease may be common in rabbits, but difficult to recognise, and lop-ears have previously been indicated as a risk factor for ear disease. We aimed to better understand the range of ear conditions in pet rabbits, signalment risk factors, and impacts on welfare and behaviour. Through an online questionnaire, we investigated owner-reported signalment, veterinary diagnosis of ear conditions, impaired hearing, and ear pain for UK pet rabbits. Relationships between ear condition measures and ear conformation, quality of life, and behaviour were analysed using logistic regression. Of 551 valid responses, 28.5% of rabbits reportedly had experienced ear conditions; 21.2% diagnosed or mentioned by vets, with otitis and excess cerumen most common. Approximately 25% of lop-eared rabbits had ear conditions indicated by a vet versus 10% of erect-eared rabbits. Lop-eared, half-lop, and older rabbits were most at risk (P<0.050). Rabbits reported as showing ear pain responses had reduced owner-reported quality of life compared with other rabbits (P<0.050). Rabbits with ear problems were less likely to be responsive to relevant sounds, and performed binky behaviour (joy jumps) less frequently, than rabbits without such issues. Understanding prevalence and risk factors for ear conditions is critical to improving welfare standards across this widely owned pet species. The findings suggest that improved recognition and treatment of ear conditions, and avoiding breeding from rabbits with early signs, or a family history, of ear disease are necessary to help combat this animal welfare issue.

## Introduction

Ear conditions could represent considerable animal welfare issues, because they can variously cause pain, hearing impairment, loss of balance, or a combination of these. We aimed to explore the nature and scale of ear conditions as a welfare issue in pet rabbits, via an owner questionnaire. In particular, we were interested in the relative prevalence of different ear conditions in a pet population, effects of ear conformation and other signalment factors on ear disease prevalence, and effects of ear conditions on rabbit welfare and behaviour.

### Prevalence of ear conditions in rabbits

Textbooks often describe ear diseases as ‘common’ in rabbits [e.g. 1–3], but prevalence estimates vary. Analysis of the first opinion clinical records of 2506 rabbits in the UK suggested a 1-year period prevalence of approximately 1.0% of rabbits having ‘auditory conditions’, as well as 1.6% having head tilts, which can signify otitis media or interna (middle or inner ear infection, respectively) [4]. Another retrospective study of clinical records of 1152 rabbits visiting a USA teaching hospital over a 20 year period found a lifetime prevalence of 3.5% of rabbits having otitis externa, 1% having otitis media, and 2% having ear mite infestation [5].

However, the true prevalence could be higher, because many ear conditions are difficult to recognise in rabbits [6, 7]. This is because behavioural signs of pain and hearing loss usually manifest as unresponsiveness and reduced activity, which can easily go unnoticed by owners or vets, especially if chronic [7–10], and rabbits may hide pain when humans are present [11]. Moreover, diagnosis of many ear conditions requires expensive, technically complex equipment, such as radiology or CT scanning for otitis media/interna, or brainstem auditory evoked response (BAER) testing for hearing loss, with some of these techniques requiring sedation of rabbits [2, 10, 12]. Nevertheless, some evidence of a higher prevalence comes from post-mortem inspection of 583 farmed adult rabbits reported in 1977, which revealed that 32% had otitis media, even though all the rabbits had appeared healthy on ante-mortem inspection [13]. More recently, retrospective examination of CT scans showed that 22% of 161 rabbits attending a UK university hospital had otitis media [14]. Notably, 61% of those cases had not been presented or referred for ear disease, suggesting that the disease was clinically ‘silent’, previously going undetected by both the owners and the referring veterinary surgeons. Similarly, another retrospective CT scan analysis revealed that 27% of 67 rabbits in the USA, again without clinical signs of ear disease, in fact had middle ear abnormalities [15]. It is worth noting that the opposite also occurred in that study, with 57% of 21 rabbits who did have clinical signs of ear disease showing no evidence of middle ear abnormalities, but perhaps this is not surprising because not all ear diseases affect the middle ear.

### Effect of ear conformation on risk of ear disease

Ear conditions have been reported as especially common in rabbits with ‘lop’ ears (pinnae that flop downwards, so the rabbit cannot move them fully or hold them erect). This is mainly because the ear canal in lop-eared rabbits is usually stenotic (narrowed) and the pinna folds over the canal, blocking cerumen (ear wax) and other exudates from being expelled [1, 16, 17]. It has also been speculated that brachycephalic skull conformations could exacerbate the problem, but this has not been directly investigated [16]. Veterinary textbooks highlight lop-eared rabbits as seeming particularly prone to otitis externa [17], otitis media and interna, excess cerumen [6], and ear-base swellings [1]. Middle ear abnormalities as identified retrospectively via CT scans were significantly more common in lop-eared rabbits than erect-eared rabbits in two separate studies [14, 15]. Furthermore, in a study of 15 lop-eared rabbits versus 15 erect eared rabbits in a rescue centre, the lop-eared rabbits showed significantly more aural problems both upon direct examination of the rabbits, and upon examination of their clinical records [16]; the conditions reported included ear canal stenosis, inflammation, cerumen, and pain responses to palpation. Conversely, no differences in the number of fungal DNA sequences were found in material from the ear canal between lop-eared and erect-eared rabbits, although results for only two breeds of each ear conformation were reported [18]. The potential association between ear conformation and risk of ear disease is concerning, because lop-eared rabbits are very popular in some countries [despite more people preferring the appearance of erect-eared rabbits: 19]. For example, between 36 and 58% of UK pet rabbits are reported as lop-eared [20–22], and at least nine recognised breeds of lop-eared rabbits exist [23].

### Effect of ear disease on welfare

The effects of ear conditions on quality of life (QoL) in animals generally are not well documented, but vets and animal welfare experts have highlighted ear disease as a potential welfare concern for pet rabbits [24]. Vets often describe ear mite infestation as intensely pruritic (itchy) [e.g. 6, 25], and even painful [7]. Few, if any, sources explicitly mention pain as being caused by otitis media or interna in rabbits, which is surprising because pain is a severe and key component of these conditions in humans [26–28]. Regarding hearing impairment, vets suggest that this could occur with excessive cerumen or waxy exudate [e.g. 6, 25], and with otitis media/interna [25, 29]. Signs can include a lack of Preyer reflex (i.e. failure to orientate the pinnae towards sound sources), but hearing loss is difficult to diagnose and quantify without BAER testing [10].

Mammals share many of the ear pathologies observed in humans [30, 31] and, in humans, hearing impairment and ear disease can greatly reduce quality of life [32, 33]. Human ear conditions as a cause of pain are common and have numerous pathological causes [34], although ear conditions not associated with pain can also impact quality of life [e.g. tinnitus 35]. Human hearing loss can cause depression [36] and anxiety [37], which could also be true for rabbits. Animals with impaired hearing are assumedly at increased risk of predation or injury, and will have a reduced ability to communicate with other individuals, both con- and hetero-specifics. Despite the likelihood that many ear conditions affect QoL in animals, we found remarkably little literature describing this. If such conditions do appreciably impair QoL in rabbits, it will be important to raise awareness of this, especially given the risk of ear conditions like otitis media going unnoticed by owners and vets. It is worth noting that QoL, when reported by owners, is a subjective measure and may not be as accurate as more objective measures of QoL. In cats and dogs, owner-reported QoL correlates in the expected direction with veterinarian assessed disease severity ratings [38, 39], although levels of agreement can still be low, because owners sometimes judge their pets as having better QoL than vets would judge them to have [40]. Nevertheless, obtaining objective measures of rabbit QoL in the home is currently extremely challenging for large scale data collection, and owner reported QoL remains a feasible and relevant metric in an assessment of domestic animal welfare and offers insight into owner recognition of animal suffering.

### Effect of ear disease on behaviour

Effective owner identification of problems to then enact interventions is critical to improving welfare for pet species. Some ear problems do cause distinct signs that may be observed by owners, according to veterinary texts. For example, moderate–severe otitis externa, including ear mites, can cause head shaking and ear-scratching by rabbits, and excess white exudate, brown crusting, and erythema (redness) may be seen inside the ear [1, 41] and can sometimes present with a swelling at the base of the ear. Similarly, otitis interna is the most common cause of head tilt and loss of balance [1]. However, otitis media is often clinically silent [13–15], with affected rabbits only occasionally showing increased head shaking or scratching [3]. Recurrent or chronic otitis media can manifest as inactivity or unresponsiveness, which can go unnoticed, or may cause non-specific signs including reduced appetite and weight loss [1].

It will be important to better understand signs of ear conditions, how owners in everyday situations perceive ear pain (e.g. via rabbits’ responses to the face and ears being touched), and hearing loss (e.g. via responsiveness to significant positive and negative sounds), as well as what they notice when looking inside their pets’ ears. Similarly, understanding of whether ear conditions reduce positive behaviours, specifically playing, binkying (‘joy jumps’ or ‘freudensprung’), and exploration, will help characterise the potential welfare outcome of ear conditions. For example, reduction of play is included within an otitis media pain scoring system for children [42], and because play is not strictly essential to survival, it is considered a ‘low resilience’ behaviour [43, 44], and a reduction of play seems to be a sensitive indicator of reduced welfare in animals [45, 46].

### Aims and hypotheses

We aimed to describe the range of ear conditions reported by owners in a population of UK pet rabbits, and to investigate whether the lop-eared phenotype increases the risk of ear problems in these rabbits. We hypothesised that, if lop-eared conformations predispose rabbits to ear conditions, then owners of lop-eared rabbits would be more likely than owners of erect eared rabbits to report (a) that a veterinarian had indicated an ear condition in their rabbit; (b) that their rabbit showed ear pain responses (behaving as if it was painful when owners looked inside their ears); (c) that their rabbit had impaired hearing or deafness; and (d) that their rabbit had an ear problem that reduced its QoL.

We also aimed to understand the potential welfare impact of ear conditions on rabbits (regardless of their ear conformation). Our hypothesis was that, if ear problems impair QoL in rabbits and if owners are able to perceive this, then rabbits reported to have veterinary indicated ear conditions, ear pain responses, or impaired hearing/deafness, would have significantly worse owner reported QoL ratings than rabbits without those problems.

Finally, we aimed to elucidate what owner-observed rabbit behaviours may be associated with ear problems including hearing impairment and ear pain responses. Awareness of these behaviours could aid owners, vets and breeders to recognise ear problems and their impact, potentially increasing treatment of these conditions in rabbits. We hypothesised that, if hearing impairment and pain affects behaviour that owners can perceive, then, compared with healthy rabbits, affected rabbits would be less responsive to sounds of a treat being prepared, less responsive to loud or threatening sounds, and less likely to perform binky behaviour, show reduced play behaviour, reduced exploration of new areas, and show avoidance of being stroked on the face (risking touching the ears).

Taken together, this information could be used to improve rabbit welfare by raising awareness of ear conditions, which rabbits may be most susceptible to ear problems, and the extent of their impact on rabbit welfare and behaviour. Increased awareness of these factors should help owners and veterinarians to identify which rabbits need treatment, and help owners and breeders to select healthy rabbits that may be less prone to debilitating ear conditions.

## Methods

### Survey creation and distribution

An online questionnaire was hosted by Survey Monkey^®^ (See S1 File for the full questionnaire wording). Before release, the questionnaire was piloted on five experienced rabbit owners, and their feedback was used to improve it. The questionnaire received ethical approval from the Royal Veterinary College’s Social Sciences Research Ethical Review Board (URN SR2021-0167), and it was live between 1^st^ November 2021 and 31^st^ January 2022. It was distributed to rabbit-owning contacts of the authors via email, more widely via social media (Facebook^™^ and LinkedIn^®^), as well as via relevant organisations including the Rabbit Welfare Association and Fund, RSPCA, the Rabbit Residence Rescue, and the Royal Veterinary College External Relations Team. It was also posted to a number of rabbit-related Facebook^™^ groups.

The anonymous respondents had to be at least 18 years old, and to currently own a rabbit. If they owned more than one rabbit, they were asked to answer the questionnaire about only one of their rabbits. To help minimise sampling bias towards or against owners of rabbits with ear disease or with particular ear conformations, the only stated aim of the project was ‘to understand how both good and bad ear health and hearing impairments affect rabbit welfare and overall quality of life’. We called for ‘owners of rabbits, whether the rabbit has excellent hearing or seems to have sore ears or difficulty hearing’. Ear conformation was not mentioned in the survey introduction, and only appeared as a signalment question in the ‘About your rabbit section’, to try to avoid leading owners to think about any connection between ear conformation and disease.

### Survey structure and content

The questionnaire took approximately 10 minutes to complete, with most questions being optional. There were 32 questions, comprising multiple choice options where respondents could either tick one answer only, or all that applied, as appropriate. There were also optional free-text comment boxes for many questions. For answer options with no natural order, e.g. lists of rabbit breeds or ear diseases, the order in which the options appeared was randomised for each respondent. This was done to help minimise order effects arising from respondents selecting early answer options more frequently than later options.

The survey was structured in sections as follows.

- Introduction. This explained the broad aim of the project as being to understand the rabbit welfare impact of ear health, asked for rabbit owners aged 18 years or over to respond, and asked for consent.
- About you. This contained demographic questions, including respondent age, gender, country of residence, and whether they worked with rabbits or in the veterinary profession.
- About your Rabbit. This contained demographic questions about the rabbit, specifying that owners must only answer about one rabbit. Questions covered the number of rabbits owned, sex and neuter status of the focal rabbit, age, breed type, and ear conformation (lop-eared, erect-eared, ‘one ear up, one down’, intermediate (approximately horizontal), or unsure).
- Your rabbit’s quality of life. In this section, owners were asked to rate their rabbit’s QoL (Very good–Very Bad) and whether the rabbit had an ear problem or other health problem that affected this. They were also asked about their rabbit’s behaviour, including response to being stroked on the face, frequency of play behaviour, binkying, response to new environments, and a subjective description of the emotional character of the rabbit (e.g. happy, fearful, and/or curious).
- Your rabbit’s hearing. This included questions about the owner-perceived extent of hearing ability in the focal rabbits, why owners believed their rabbit could/could not hear well, and how their rabbit responded to specific sounds (owner approach, opening a treat packet, and loud or threatening sounds).
- Your rabbit’s health. Questions in this section included frequency of ear cleaning by a vet, questions about what was seen in the rabbit’s ears and how the rabbit responded to the owner looking into its ears, ear conditions indicated by a vet, and age of diagnosis. A final invitation for any other comments as free-text was also provided.

### Criteria for exclusion

Respondents were excluded if they reported being under 18 years old or did not provide their age, or who did not live in the UK (due to the fact that insufficient responses were received from outside the UK). Any respondents who dropped out without answering the ‘about your rabbit’ section, or before answering any questions in the subsequent sections, were excluded. Respondents reporting not owning a rabbit, or having only owned a rabbit in the past, were also excluded. Respondents who reported current fostering of rabbits were included.

### Statistical analysis

Following manual data check and cleaning, analyses were performed using SPSS (version 28.0, IBM Corp, NY, USA). Some respondents did not answer all the questions, so the sample size varied between analyses. Categorical data were described as percentages (of the number of responses for that question, including ‘Unsure’) and non-normally distributed scales were summarised using median and interquartile range (IQR). Some variables could not be analysed due to insufficient variation in responses (e.g. respondents very rarely described their rabbits as ‘worried’, ‘stressed’ or ‘angry’). A Kruskall-Wallis H test was used to compare the distribution of rabbit age across the ear conformations. Pearson’s chi-square tests with pairwise Z-tests were used to compare the distribution of sex/neuter status, owner age, and owner gender between erect and lop-eared rabbits. To test the convergent validity of our four measures of ear problems, Mann-Whitney U tests were performed to understand relationships between each pair of ordinal and binary variables.

To investigate risk-factors for ear or QoL outcomes, multivariate binary logistic regressions were performed. Individual models were produced for each outcome variable and included other background predictor variables, as detailed below. Outcome variables with more than two categories were collapsed to form binary variables (see below and S2 File). Categories that comprised fewer than 15 data points in the entire sample, and that could not be meaningfully combined with other categories, were excluded from models. The logistic regression models were checked for inflated standard errors of the estimates. Where inflated standard errors occurred, either the rarest categories within the affected variable were collapsed or removed and the adjusted variable reinserted into the model; or, if this did not help, the affected variables were removed and tested within alternative models without conflicting variables. Logistic regression model effects were reported as odds ratios (OR) and confidence intervals (CI: 95% lower—upper) with significance set at *P* < 0.05.

To investigate whether ear conformation predicted ear disease, separate logistic regression models were created with the collapsed, binary outcome variables of: owner reported veterinary indication of an ear problem (with ‘indication’ referring to combined responses of ‘mentioned, but not diagnosed’ and ‘yes, diagnosed’ from the question ‘Has a vet ever diagnosed your rabbit as having any ear problem(s)?’); hearing impairment; ear pain response (from the question: ‘Do you think your rabbit behaves as if it is painful when you try to look into their ears?’); and an ear-related problem which affected rabbit QoL. Predictors used in these models were ear conformation, rabbit age, sex/neuter status, owner age, and owner sex. The two-way interaction between age and ear conformation was also initially included in the models and removed if non-significant.

To test whether owners perceived ear conditions to affect rabbit QoL, reported overall QoL was the outcome variable and was collapsed into two categories: ‘very good’, and ‘reduced’, which incorporated all other responses (which comprised ‘good’ or ‘moderate’). Further output variables tested were the owner reports of their rabbits being ‘happy’, ‘relaxed’, ‘curious’, or ‘nervous/fearful’. Predictor variables tested in separate models were non-collapsed reports of hearing ability, and the binary conditions of vet indication of an ear problem, and having ear pain response or not. Additional predictors used in this model were again rabbit age, sex/neuter status, owner age, and owner sex, plus whether the rabbit had a non-ear-related health condition that impaired their QoL.

To investigate perceived behavioural predictors of ear problems, outcome variables of ‘does not respond, and may startle, when approached from behind’, ‘does not respond to the sound of a treat (unless they can see/smell the treat)’, ‘does not respond to loud or threatening sounds’ were each collapsed into binary variables indicating responsiveness or unresponsiveness to the respective sounds. Further binary outcome variables of a rabbit ‘does not enjoy being stroked around the face/ears’ (At least sometimes/Not particularly or no), ‘rarely performs binky behaviour’ (At least sometimes/Rarely), ‘plays with toys, other rabbits, or familiar people?’ (Sometimes/Not particularly), and ‘actively explores new environments’ (explorative/cautious) were also modelled. Predictor variables included non-collapsed vet indication of an ear problem and impaired hearing, and the binary condition of having ear pain response. Additional predictors used in these models were ear conformation, rabbit age, sex/neutered status, owner age, owner sex.

## Results

### Demographics

Initially, 693 responses were received, with 142 responses being excluded by the defined criteria. Of the 551 included respondents, 95.1% were female and 4.0% were male. Four respondents were non-binary (0.72%) and a single person selected ‘prefer not to say’. The most common age category was 25–34 (37.0%), followed by 35–44 (22.7%). The younger, 18–24 (13.7%) and older age groups, 45–54 (15.6%), 55–64 (8.5%) and 65+ (2.4%), were less represented. The oldest two age categories were combined for analysis due to the low numbers. Regarding work type, 238 (75.6%) of respondents did not work with rabbits, and 35 (11.1%) reported working with rabbits (e.g. rescue, charity, or breeder). There were 15 (4.8%) vet students and 11 (3.5%) veterinarians. Almost half of all respondents (46.7%) owned two rabbits, with 31.8% owning a single rabbit and 21.0% owning three or more.

Of the 551 rabbits, 48.5% of rabbits were reported as being lop-eared, 42.5% were erect-eared, and the remainder were either asymmetrical (‘half-lop’; one erect ear and one lop), or had approximately horizontal ears (‘oar-lop’ or ‘horn-lop’; Table 1). Median rabbit age was 4 years (IQR: 2–6) and this was not significantly different across ear conformations (*P* = 0.314). There was similarly no significant difference in the proportion of sex/neutered status (*P* = 0.880), owner age (*P* = 0.764), or owner gender (*P* = 0.692) between the erect and lop-eared rabbits. The most common owner reported breed types were dwarf/mini lops (which were a combined category in the survey) and crossbreeds.

**Table 1:** Rabbit signalment for each ear conformation.

Descriptive category	Signalment and ear-related problems	Ear conformation				Total
		Erect	Lop	Asymmetrical	Horizontal	
Total rabbits (n)	n/a	234	267	32	18	551
Age (Median (IQR) years)	n/a	4 (2–6)	4 (2–6)	4 (2–6)	2 (1–6)	4 (2–6)
Neuter status (n)	*Female neutered*	85	90	12	6	193
	*Female entire*	11	15	1	3	30
	*Male neutered*	117	140	13	7	277
	*Male entire*	21	22	6	1	50
Ten most reported breed types (n)	Dwarf lop/mini lop	0	154	6	1	161
	Crossbreed	75	32	15	5	127
	Netherland dwarf	35	0	0	3	38
	Lionhead	32	0	3	2	37
	Dutch	21	2	0	0	23
	French lop	0	20	0	0	20
	English lop	0	17	1	0	18
	Rex	16	1	0	1	18
	Mini Lionhead-lop	0	12	0	0	12
	Continental Giant	9	0	0	0	9

Breeds are arranged from most to least common in total. All percentages are calculated from the sub-total of respondents who answered each question, where this differs from the total number of respondents.

### Description of ear disease and convergent validity

Owners reported 102 rabbits (21.2%) as having had a vet indicated ear problem (Table 2). Taken together, 123 (28.5%) of the 432 rabbits with complete data for all four ear condition measures (vet indication, ear problem affecting QoL, pain response, and/or hearing impairment) were reported to have had at least one of these conditions; the median number of these four ear conditions per affected rabbit was 2 (IQR = 1–3). Of these affected rabbits, 24.1% had never received a veterinary indication of their ear condition. Owners reported that 34 (7.1%) of all the rabbits had a perceived pain response when owners looked in the ears, 83 (15.8%) had impaired hearing, and 75 (13.6%) had an ear-related problem that reduced rabbit QoL. In free-text comments, two rabbits who were seemingly unaffected according to our four measures of ear problems, were reported to frequently scratch at their ears.

**Table 2:** Number of reports for ear-related problems diagnosed or mentioned by a vet for each ear conformation.

Descriptive category	Signalment and ear-related problems	Ear conformation				Total
		Erect	Lop	Asymmetrical	Horizontal	
Presence of vet indicated ear problem (n (%))	n/a	23 (11.3)	69 (29.7)	9 (32.1)	1 (6.7)	102 (21.2)
*Specific vet indicated ear related problems (n (%))*	Any form of Otitis	10 (5)	40 (17.2)	6 (21.4)	0	56 (11.7)
	Excessive ear wax (cerumen)	4 (2.0)	20 (8.6)	3 (10.7)	0	27 (5.6)
	Middle ear infection (Otitis media)	4 (2.0)	15 (6.5)	2 (7.1)	0	21 (4.4)
	Inner ear infection, vestibular disease or labyrinthitis (Otitis interna)	2 (1.0)	17 (7.3)	2 (7.1)	0	21 (4.4)
	Unspecified ear infection (Otitis)	3 (1.5)	12 (5.2)	3 (10.7)	0	18 (3.8)
	Inflammation or infection of the outer ear (Otitis externa)	2 (1.0)	12 (5.2)	1 (3.6)	0	15 (3.1)
	Ear mites	7 (3.5)	9 (3.9)	0	0	16 (3.3)
	Hearing loss	2 (1.0)	13 (5.6)	1 (3.6)	1 (6.6)	16 (3.3)
	Ear abscess	1 (0.5)	9 (3.9)	0	0	10 (2.1)
	Cut or other injury to the outer ear	0	1 (0.4)	0	0	1 (0.2)
Ear pain responses (n (%))	Yes	5 (2.5)	27 (11.7)	2 (7.1)	0	34 (7.1)
	No	184 (91.1)	191 (82.7)	23 (82.1)	13 (86.7)	411 (85.8)
Hearing (n (%))	Very good	147 (66.8)	79 (31.5)	15 (46.9)	8 (44.4)	249 (47.4)
	Good	66 (30)	91 (36.3)	15 (46.9)	8 (44.4)	180 (34.2)
	Impaired	4 (1.8)	46 (18.3)	1 (3.1)	0	51 (9.7)
	Deaf	2 (0.9)	27 (10.8)	1 (3.1)	2 (11.1)	32 (6.1)

Specific ear-related problems are arranged from most to least common in total. Multiple specific ear problems could be selected for the same rabbit. All percentages are calculated from the sub-total of respondents who answered each question, where this differs from the total number of respondents.

The most common ear conditions indicated by a vet were otitis–particularly otitis media and interna–and excessive ear wax (Table 2); ear mites and hearing loss were also reported to have been indicated in a minority of rabbits. Median initial age of vet indication of an ear condition was 3 (IQR = 2–5) years, with this occurring at 3 years or younger in 48.3% of rabbits who were diagnosed with such issues. Owners reported that when they looked inside their rabbits’ ears, the majority observed healthy skin, fur, and/or the ear canal (S3 File). The most commonly observed problems were a small amount of ear wax, the rabbit flinching, one or more small bumps or spots, or the ear canal not being visible. The hole of the ear canal was reported not to be visible in 12.1% of rabbits who had an ear problem indicated by a vet compared with 2.4% of those without such indication (S3 File).

Regarding convergent validity of our four measures of ear problems, ear pain responses, hearing ability, and QoL were all worse in rabbits with vet indicated ear problems. Higher ear pain response scores were more prevalent in rabbits with vet indicated ear problems (pain score median = 3, IQR = 3–3) than without such vet indication (pain score median = 3, IQR = 2–3, *U* = 21554, N = 445, *P* < 0.001). Of the rabbits with a vet indicated ear problem, and whose owners had looked into their ears, 11.1% were reported to flinch and pull away during ear examination compared with 2.6% of rabbits flinching during ear examination if they had no such veterinary indication. Similarly, lower hearing ability scores (indicating more impaired hearing) were more prevalent with vet indicated ear problems (median = 3, IQR = 2–3) than without (median = 4, IQR = 3–4, *U* = 28037.5, N = 471, *P* < 0.001). Finally, better QoL scores were more prevalent without vet indicated ear problems (median = 2, IQR = 2–2) than with such indications (median = 1, IQR = 1–2, N = 470, *U* = 27705, *P* < 0.001).

### Lop-eared phenotype and other signalment as risk factors for reported ear problems

**Fig 1:**
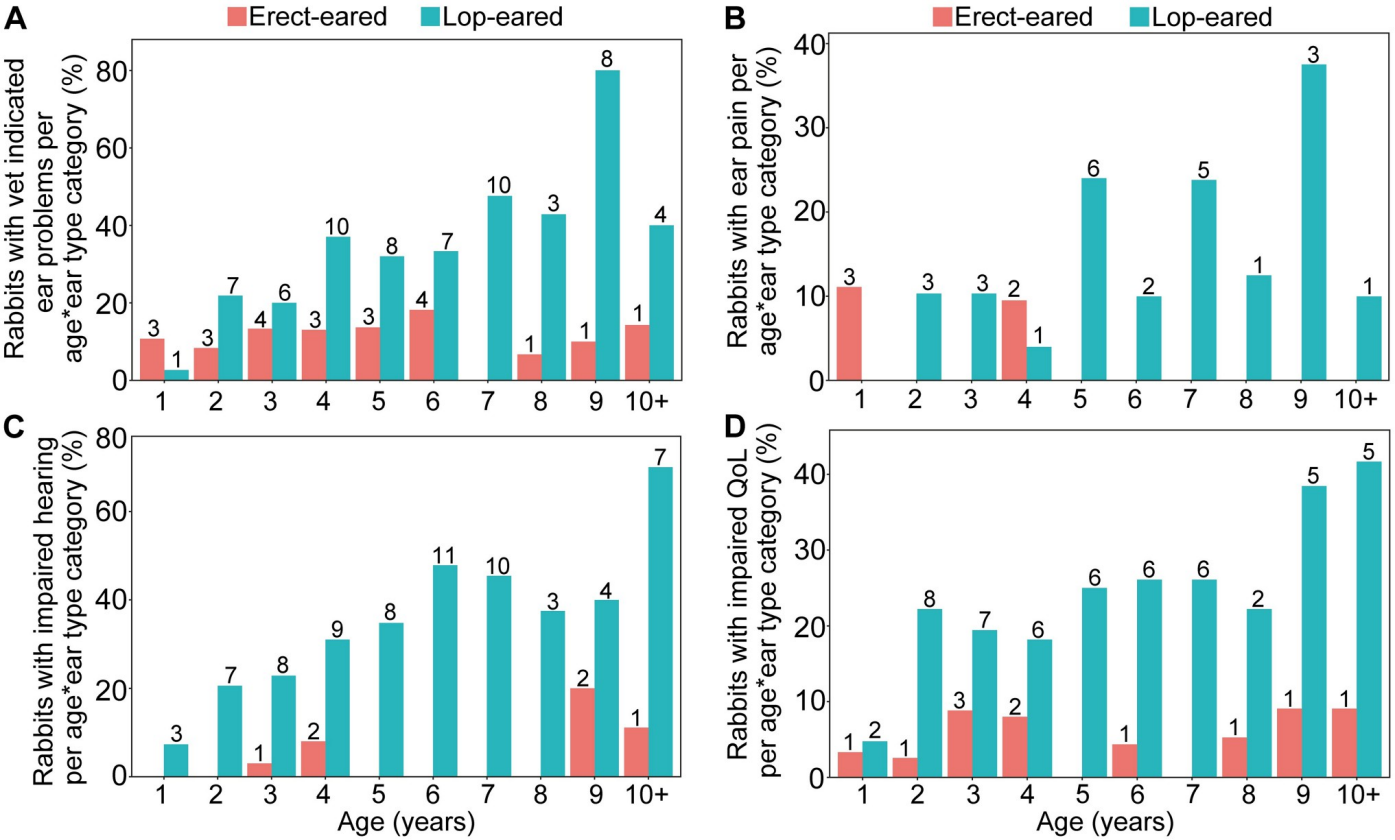
Reported ear problems for each age category for the lop-eared and erect-eared rabbits. Modelled ear conditions are (A) vet indication of an ear condition, (B) ear pain responses, (C) impaired hearing, and (D) impaired quality of life. Erect-eared rabbits are shown in red, and lop-eared rabbits in blue. Numbers on bars represents total rabbits for that category of ear conformation and age.

One quarter (24.7%) of the lop-eared rabbits in this survey population had a vet indicated ear problem, compared with 9.8% of erect-eared rabbits, and almost all specific conditions reported were seemingly more common in the lop-eared phenotype than in the other ear conformations (Table 2 and S3 File). Logistic regression modelling suggested statistically significant two-way interactions, such that vet indicated ear problems (*P* = 0.048) and ear pain responses (*P* = 0.024) increased with age, but only for the lop eared rabbits and not the other ear conformations (Table 3; Fig 1). Lop eared rabbits were ~19 times more likely to be reported as having impaired hearing or deafness than erect eared rabbits were (*P* < 0.001), with increasing age having an independent worsening effect (*P* <0.001). The interaction between ear conformation and age was not significant for hearing impairment or deafness. Also, compared with erect eared rabbits, lop eared rabbits were 5.5 times more likely to be reported as having impaired QoL due to an ear problem (*P* < 0.001), and asymmetrical-eared rabbits 4.1 times more likely (*P* = 0.019). Older age was also associated with reduced QoL across rabbits of all ear conformations (*P* < 0.001; Table 3; Fig 1). Additionally, men were more likely than women to report ear pain responses in their rabbits (Table 3). No other rabbit or owner factors reached statistical significance in the models.

**Table 3:** Statistically significant binary logistic regression results for ear-related issues for the interaction of rabbit age and ear conformation.

Outcome variable	Model fit	Statistically significant predictor variables and terms	N for individual categories (%)	Effect size	*P*-value
Vet indicated ear problem (n = 458)	χ^2^(15) = 57.49, P < 0.001	Ears * Age	n/a	Wald: 9.24	0.026*
		> Age * Erect	200 (43.7)	Reference	Reference
		> Age * Lop	218 (47.6)	B: 0.28, SE: 0.11, Wald: 6.77,	0.009*
		> Age * Asymmetrical	26 (5.7)	B: -0.05, SE: 0.19, Wald: 0.07,	0.787
		> Age * Horizontal	14 (3.1)	B: 0.77, SE: 0.84, Wald: 0.84,	0.359
Reported ear pain responses (n = 394)	χ^2^(11) = 29.26, P = 0.002	Ears * Age	n/a	Wald: 5.07	0.024*
		> Age * Erect	187 (47.5)	Reference	Reference
		> Age * Lop	207 (52.5)	B: 0.72, SE: 0.32, Wald: 5.07,	0.024*
		Owner gender	n/a	Wald: 3.92	0.048*
		> Female	380 (96.4)	Reference	Reference
		> Male	14 (3.6)	OR: 4.56, CI: 1.02–20.50	0.046*
Reported impaired hearing (n = 493)	χ2(12) = 107.04, P < 0.001	Ears	n/a	Wald: 46.81	< 0.001*
		> Erect	215 (43.6)	Reference	Reference
		> Lop	231 (46.9)	OR: 18.52, CI: 7.62–45.02	< 0.001*
		> Asymmetrical	30 (6.1)	OR: 2.73, CI: 0.5–15.04	0.248
		> Horizontal	17 (3.4)	OR: 3.19, CI: 0.34–29.70	0.309
		Age	n/a	B: 0.28, SE: 1.19–1.48, Wald: 0.06,	< 0.001*
Impaired QoL (n = 517)	χ2(12) = 52.86, P < 0.001	Ears	n/a	Wald: 23.90	< 0.001*
		> Erect	224 (43.3)	Reference	Reference
		> Lop	247 (47.8)	OR: 5.48, CI: 2.75–10.99	< 0.001*
		> Asymmetrical	30 (5.8)	OR: 4.07, CI: 1.26–13.20	0.019*
		> Horizontal	16 (3.1)	OR: 1.57, CI: 0.1 8–13.53	0.680
		Age	n/a	B: 0.14, SE: 0.80, Wald: 7.75	0.005*

Models also included rabbit age, sex/neutered status, owner age and owner gender, as well as age and ear conformation separately. Interactions are not included if non-significant, in which case the individual terms are reported. For the interaction of age and ear conformation in relation to reported ear pain responses, asymmetrical and horizontal eared rabbits were excluded due to inflated standard errors in the model. Odds ratios are given in relation to the reference category indicated.

*Only variables with significant effects (P < 0.050) are reported.

B: coefficient, SE: standard error, OR: odds ratio, CI: 95% confidence intervals. QoL: quality of life in relation to a hearing condition.

### Reported ear problems and overall QoL

**Fig 2:**
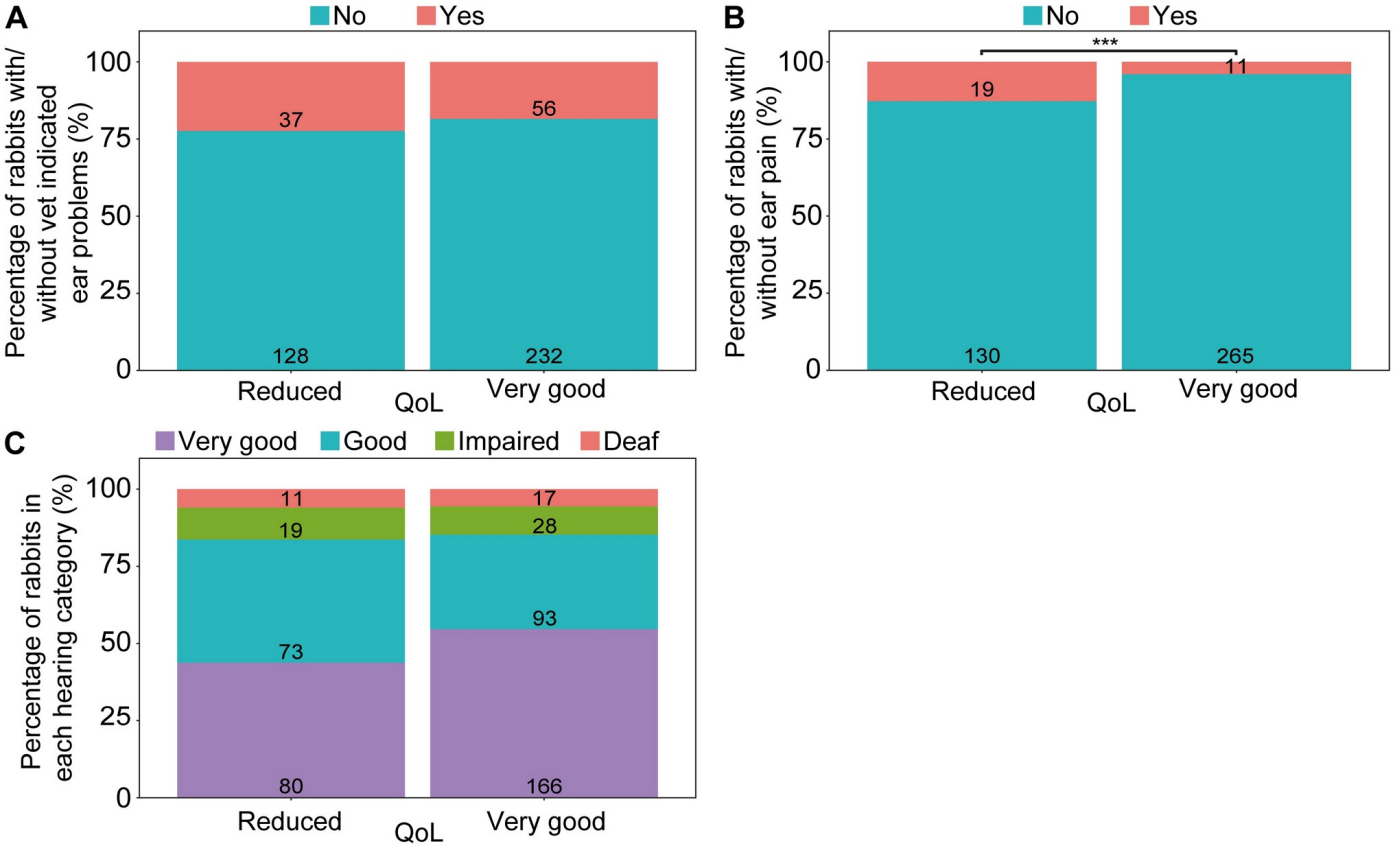
Reported ear problems across the categories of QoL for all rabbits. Numbers on bars represent total rabbits for that category. *** P ≤ 0.001.

In total, 75 (13.6%) respondents reported their rabbit as having an ear problem that reduced QoL; conversely 6.0% reported their rabbit to have an ear problem that did not affect their QoL. When controlling for cases where QoL was affected by a non-ear problem, rabbits reported to have ear pain responses were three times more likely to have a perceived reduction in overall QoL than those without ear pain responses (*P* = 0.010; Fig 2; Table 4). Conversely, rabbits being reported as having had a vet indicated ear problem or having impaired hearing, did not significantly predict overall QoL (Fig 2). In all cases, rabbits reported as having non-ear-related conditions that affected their quality of life were indeed more likely to be reported as having reduced QoL than those without such conditions (*P* <0.001). Regarding owners’ subjective descriptions of their rabbits’ temperament, rabbits reported to have an ear problem indicated by a vet were significantly more likely to be reported as happy than those without a vet indication (*P* = 0.038; Table 4). Owner reports of rabbits being nervous/fearful, relaxed, or curious did not significantly differ between the groups of ear pain responses, vet indication, or hearing impairment.

**Table 4:** Statistically significant logistic regression results for predictors of reduced QoL and reported emotional state.

Outcome variable	Predictor variable	Model fit	Statistically significant predictor variables and terms	N (%)	Effect size	*P*-value
Reduced QoL	Vet indicated ear problem (n = 453)	χ^2^(11) = 32.42, P = 0.001	Vet indication	n/a	Wald: < 0.001	0.983
			> No	360 (79.5)	Reference	Reference
			> Yes	93 (20.5)	OR: 0.10, CI: 0.60–1.65	0.983
			Non-ear condition	n/a	Wald: 19.64	< 0.001*
			> No	361 (79.7)	Reference	Reference
			> Yes	92 (20.3)	OR: 3.21, CI: 1.92–5.38	< 0.001*
	Reported ear pain responses (n = 425)	χ^2^(11) = 31.88, P = 0.001	Ear pain responses	n/a	Wald: 6.72	0.010*
			> No	395 (92.9)	Reference	Reference
			> Yes	30 (7.1)	OR: 2.90, CI: 1.30–6.49	0.010*
			Non-ear condition	n/a	Wald: 13.14	< 0.001*
			> No	336 (79.1)	Reference	Reference
			> Yes	89 (20.9)	3.32	< 0.001*
	Reported impaired hearing (n = 487)	χ^2^(13) = 36.06, P = 0.001	Hearing	n/a	Wald: 2.62	0.455
			> Very good	246 (50.5)	Reference	Reference
			> Good	166 (34.1)	OR: 1.39, CI: 0.90–2.13	0.137
			> Impaired	47 (9.7)	OR: 0.97, CI: 0.48–1.96	0.935
			> Deaf	28 (5.7)	OR: 0.99, CI: 0.42–2.33	0.976
			Non-ear condition	n/a	Wald: 17.57	< 0.001*
			> No	386	Reference	Reference
			> Yes	101	OR: 2.89, CI: 1.76–4.76	< 0.001*
Reported as ‘Happy’	Vet indicated ear problem (n = 453)	χ^2^(11) = 19.92, P = 0.046	Vet indication	n/a	Wald: 4.31	0.038*
			> No	360 (79.5)	Reference	Reference
			> Yes	93 (20.5)	2.54	0.038*
			Owner age	n/a	Wald: 11.55	0.021*
			> 18–24	57 (12.6)	Reference	Reference
			> 25–34	171 (37.7)	OR: 2.46, CI: 1.13–5.39	0.024*
			> 35–44	101 (22.3)	OR: 2.86, CI: 1.16–7.08	0.023*
			> 45–54	75 (16.6)	OR: 1.23, CI: 0.53–2.89	0.632
			> 55+	49 (10.8)	OR: 0.96, CI: 0.38–2.42	0.934

Odds ratios are given in relation to the reference category indicated. Models also included rabbit age, sex/neutered status, owner age and owner gender.

*Only main predictor variable and variables with significant effects (P < 0.050) are reported.

OR: odds ratio, CI: 95% confidence intervals. QoL: quality of life.

### Behavioural and physical signs of ear problems

**Fig 3:**
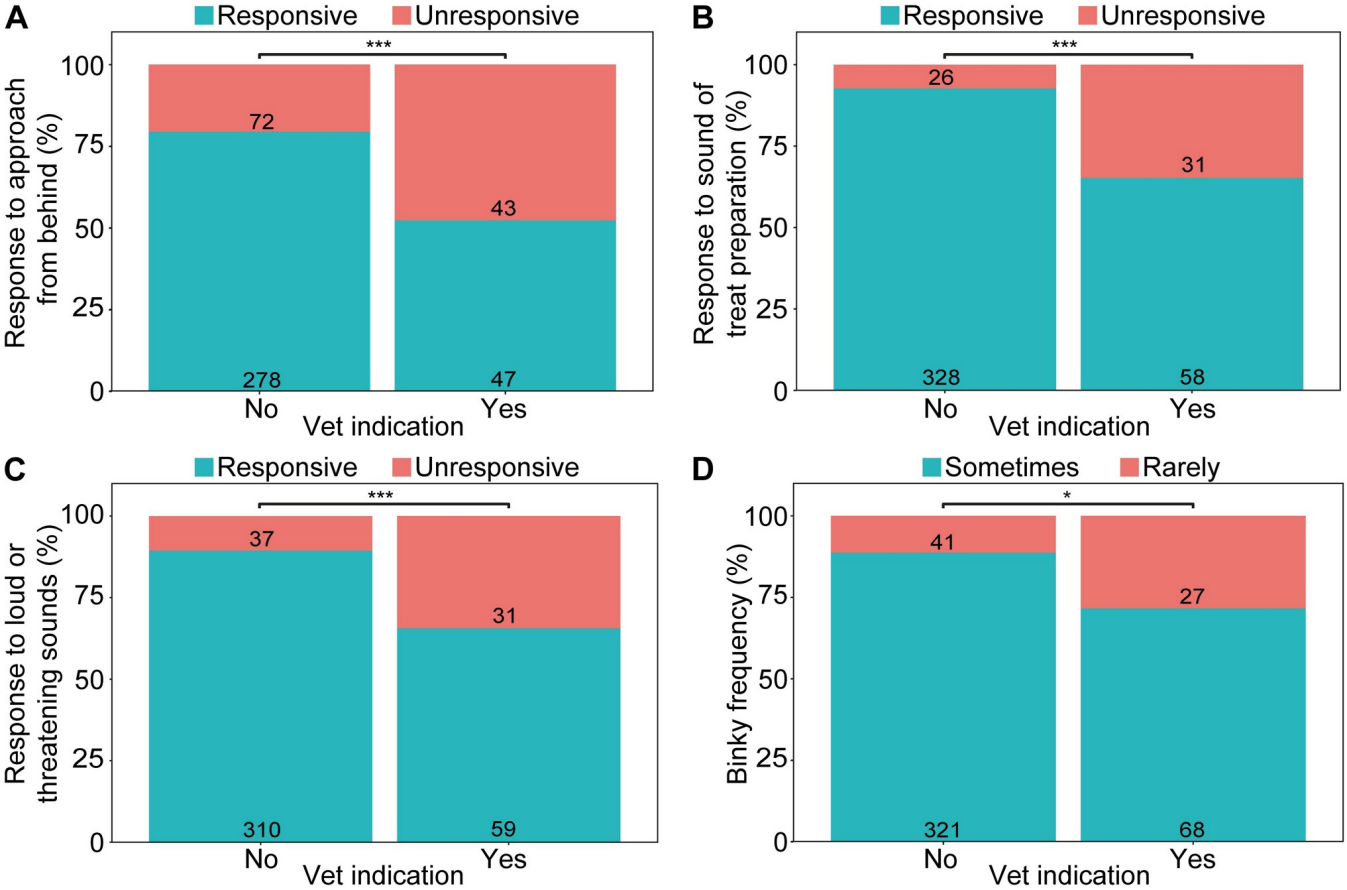
Rabbit behaviour with and without reported vet indication of ear problems. Behavioural indicators are (A) response to an approach from behind, (B) response to a treat being prepared within earshot, (C) response to loud or threatening sounds, and (D) frequency of performing binky behaviour. Numbers on bars represents total rabbits for that category. * P < 0.05; *** P ≤ 0.001.

Rabbits who had a vet indicated ear condition were more than twice as likely to be unresponsive to an approach from behind (*P* = 0.001; Table 5), a treat being prepared within earshot (*P* < 0.001), and loud or threatening sounds (*P* = 0.001) than rabbits who had had no such indication (Fig 3). Lop-eared rabbits were also more likely to be unresponsive in these situations than erect-eared rabbits (Table 5).

**Table 5:** Binary logistic regression results for rabbit behavioural indicators predicted by vet indication of an ear problem, ear pain responses, and hearing impairment.

Predictor variable	Outcome variable	Model fit	Statistically significant predictor variables and terms	n (%)	Effect size	*P*-value
Vet indication of an ear problem	Unresponsive to an approach from behind (n = 440)	χ^2^(13) = 87.08, P < 0.001	Vet indication	n/a	Wald: 13.06	< 0.001*
			> No	350 (79.5)	Reference	Reference
			> Yes	90 (20.5)	OR: 2.82. CI: 1.61–4.93	< 0.001*
			Ears	n/a	Wald: 37.95	< 0.001*
			> Erect	194 (44.1)	Reference	Reference
			> Lop	209 (47.5)	OR: 4.86, CI: 2.82–8.35	< 0.001*
			> Asymmetrical	24 (5.5)	OR: 0.57, CI: 0.12–2.69	0.479
			> Horizontal	13 (3)	OR: 1.71, CI: 0.34–8.55	0.514
	Unresponsive to a treat being prepared within earshot (n = 443)	χ^2^(13) = 97.04, P < 0.001	Vet indication	n/a	Wald: 15.36	< 0.001*
			> No	354 (79.9)	Reference	Reference
			> Yes	89 (20.1)	OR: 3.97, CI: 1.99–7.90	< 0.001*
			Ears	n/a	Wald: 24.77	< 0.001*
			> Erect	197 (44.5)	Reference	Reference
			> Lop	208 (47.0)	OR: 12.83, CI: 4.39–37.51	< 0.001*
			> Asymmetrical	24 (5.4)	OR: 1.71, CI: 0.17–17.01	0.646
			> Horizontal	14 (3.2)	OR: 4.61, CI: 0.42–50.29	0.210
			Age	n/a	B: 0.227, SE: 0.07, Wald: 11.97	< 0.001*
	Unresponsive to a loud or threatening sound (n = 437)	χ^2^(13) = 59.62, P < 0.001	Vet indication	n/a	Wald: 11.70	0.001*
			> No	347 (79.4)	Reference	Reference
			> Yes	90 (20.6)	OR: 2.94, CI: 1.58–5.44	0.001*
			Ears	n/a	18.82	< 0.001*
			> Erect	192 (43.9)	Reference	Reference
			> Lop	208 (47.6)	OR: 4.29, CI: 2.14–8.60	< 0.001*
			> Asymmetrical	23 (5.3)	OR: 0.70, CI: 0.08–5.80	0.739
			> Horizontal	14 (3.2)	OR: 3.07, CI: 0.57–16.44	0.190
			Age	n/a	B: 0.15, SE: 0.06, Wald: 6.82	0.009*
	Rarely performs binky behaviour (n = 457)	χ^2^(13) = 50.97, P < 0.001	Vet indication	n/a	Wald: 9.85	0.002*
			> No	362 (79.2)	Reference	Reference
			> Yes	95 (20.8)	OR: 2.68, CI: 1.45–4.97	0.002*
			Age	n/a	B: 0.27, SE: 0.05, Wald: 20.49	< 0.001*
Ear pain responses	Unresponsive to a treat being prepared within earshot (n = 414)	χ^2^(13) = 90.94, P < 0.001	Ear pain responses	n/a	Wald: 10.36	0.001*
			> No	386 (93.2)	Reference	Reference
			> Yes	28 (6.8)	OR: 5.01, CI: 1.88–13.37	0.001*
			Ears	n/a	Wald: 24.07	< 0.001*
			> Erect	184 (44.4)	Reference	Reference
			> Lop	197 (47.6)	OR: 17.98, CI: 5.36–60.27	< 0.001*
			> Asymmetrical	21 (5.1)	OR: 2.84, CI: 0.27–30.53	0.389
			> Horizontal	12 (2.9)	OR: 7.57, CI: 0.09–3.41	0.101
			Age	n/a	B: 0.25, SE: 0.07, Wald: 14.93	< 0.001*
	Rarely performs binky behaviour (n = 429)	χ^2^(13) = 45.60, P < 0.001	Ear pain responses	n/a	Wald: 5.72	0.017*
			> No	398 (92.8)	Reference	Reference
			> Yes	31 (7.2)	OR: 3.03, CI: 1.22–7.51	0.017*
			Age	n/a	B: 0.27, SE: 1.06, Wald: 16.99	< 0.001*
Hearing impairment	Unresponsive to an approach from behind (n = 471)	χ^2^(15) = 191.73, P < 0.001	Hearing	n/a	Wald: 72.22	< 0.001*
			> Very good	233 (49.5)	Reference	Reference
			> Good	161 (34.2)	OR: 2.85, CI: 2.02–7.33	< 0.001*
			> Impaired	48 (10.2)	OR: 39.70, CI: 14.76–106.81	< 0.001*
			> Deaf	29 (6.2)	OR: 135.38, CI: 27.86–657.81	< 0.001*
			Ears	n/a	Wald: 7.25	0.064
			> Erect	208 (44.2)	Reference	Reference
			> Lop	220 (46.7)	OR: 2.17, CI: 1.17–4.03	0.014*
			> Asymmetrical	28 (5.9)	OR: 1.03, CI: 0.28–3.78	0.962
			> Horizontal	15 (3.2)	OR: 0.72, CI: 0.11–4.83	0.734
			Owner age	n/a	Wald: 12.37	0.015*
			> 18–24	63 (13.4)	Reference	Reference
			> 25–34	184 (39.1)	OR: 2.38, CI: 0.91–6.21	0.077
			> 35–44	100 (21.2)	OR: 1.87, CI: 0.65–5.41	0.245
			> 45–54	74 (15.7)	OR: 1.73, CI: 0.57–5.25	0.331
			> 55+	50 (10.6)	OR: 6.75, CI: 2.12–21.51	0.001*
	Unresponsive to a treat being prepared within earshot (n = 476)	χ^2^(15) = 231.80, P < 0.001	Hearing	n/a	Wald: 73.20	< 0.001*
			> Very good	239 (50.2)	Reference	Reference
			> Good	164 (34.5)	OR: 3.19, CI: 0.61–16.74	0.170
			> Impaired	44 (9.2)	OR: 84.17, CI: 16.18–437.93	< 0.001*
			> Deaf	29 (6.1)	OR: 1213.95, CI: 140.74–10471.15	< 0.001*
			Age	n/a	B: 0.20, SE: 0.09, Wald: 4.76	0.029*
			Owner age	n/a	Wald: 4.89	0.299
			> 18–24	64 (13.4)		Reference
			> 25–34	183 (38.4)	OR: 0.45, CI: 0.11–1.81	0.260
			> 35–44	98 (20.6)	OR: 0.38, CI: 0.08–1.78	0.220
			> 45–54	80 (16.8)	OR: 0.14, CI: 0.02–0.88	0.036*
			> 55+	51 (10.7)	OR: 0.63, CI: 0.12–3.40	0.595
	Unresponsive to a loud or threatening sound (n = 474)	χ^2^(15) = 163.29, P < 0.001	Hearing	n/a	53.02	< 0.001*
			> Very good	236 (49.9)	Reference	Reference
			> Good	163 (34.3)	OR: 1.10, CI: 0.46–2.68	0.826
			> Impaired	47 (9.9)	OR: 11.72, CI: 4.41–31.45	< 0.001*
			> Deaf	28 (5.9)	OR: 380.62, CI: 45.11–3211.62	< 0.001*
	Rarely performs binky behaviour (n = 491)	χ^2^(15) = 52.43, P < 0.001	Hearing	n/a	Wald: 7.15	0.067
			> Very good	243 (49.5)		Reference
			> Good	169 (34.4)	OR: 2.26, CI: 1.19–4.29	0.013*
			> Impaired	50 (10.2)	OR: 1.20, CI: 0.43–3.36	0.728
			> Deaf	29 (5.9)	OR: 2.12, CI: 0.70–6.38	0.183
			Age	n/a	B: 0.29, SE: 0.06, Wald: 27.01	< 0.001*

For each ear conformation, odds ratios (ORs) are given in relation to the reference category of being ‘responsive’ to sounds or more often performing the behaviour, as indicated. Only statistically significant effects are reported, but models also included ear conformation, rabbit age, sex/neutered status, owner age and owner gender.

* P < 0.05.

Rabbits who had had a vet indicated ear problem were also more likely to be reported as only rarely performing binkying behaviour than rabbits without these problems, who binkied more frequently (*P* = 0.002; Table 5). As age increased, it was significantly more likely that rabbits were reported as being unresponsive to a treat being prepared within earshot and to loud or threatening sounds, and to rarely perform binky behaviour (Table 5).

**Fig 4:**
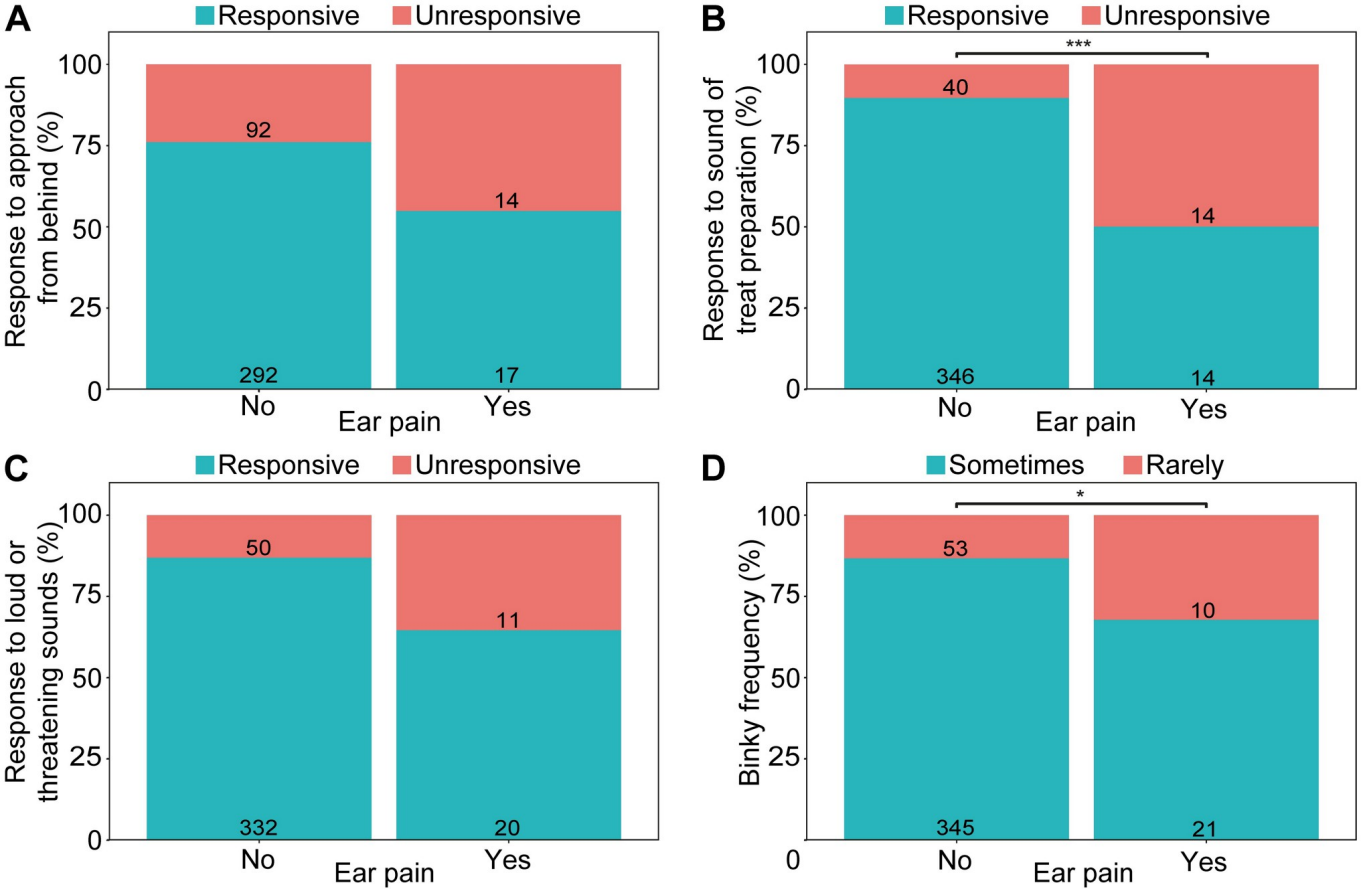
Rabbit behaviour with and without reported ear pain responses. Behavioural indicators are (A) response to an approach from behind, (B) response to a treat being prepared within earshot, (C) response to loud or threatening sounds, and (D) frequency of performing binky behaviour. Numbers on bars represents total rabbits for that category. * P < 0.05; *** P ≤ 0.001.

Rabbits reported to have ear pain responses were significantly more likely to be unresponsive to a treat being prepared behind them than those who were not (Fig 4; *P* = 0.001) and were ~3 times less likely to perform binky behaviour than those without reported ear pain responses (Fig 4; *P* = 0.017; Table 5).

**Fig 5:**
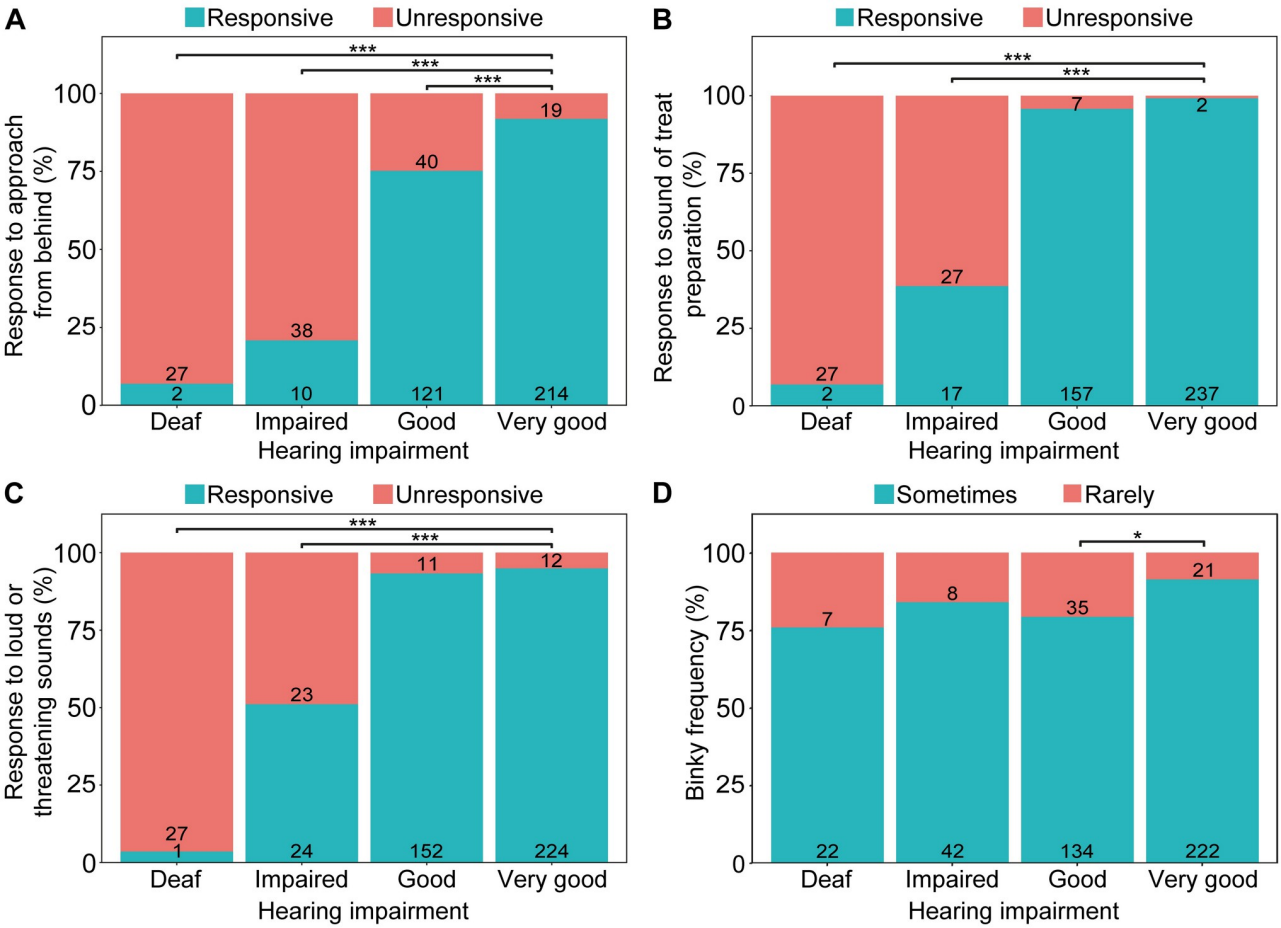
Rabbit behavioural indicators of reported hearing ability. Behavioural indicators are (A) response to an approach from behind, (B) response to a treat being prepared within earshot, (C) response to loud or threatening sounds, and (D) frequency of performing binky behaviour. Numbers on bars represents total rabbits for that category. * P < 0.05; *** P ≤ 0.001.

Rabbits reported as deaf were more likely to be unresponsive to sound cues than rabbits who were not deaf (Fig 5; Table 5; *P* < 0.001). Similarly, rabbits reported to have impaired hearing were also more likely to be reported as being unresponsive to being approached from behind (*P* = < 0.001), to having a treat prepared within earshot (*P* = < 0.001), and to loud and threatening sounds (*P* = < 0.001) than rabbits who had ‘very good’ hearing. Lop-eared rabbits were twice as likely to be reported as unresponsive to being approached from behind than erect eared rabbits (*P* = 0.014). Rabbits being reported as having ‘good’ hearing were 2.3 times more likely to be reported as rarely binkying than rabbits who had ‘very good’ hearing (*P* = 0.011).

None of the measures of ear problems significantly predicted the rabbits’ responses to being stroked around the face, the frequency of general play behaviour (other than binkying specifically), or their behaviour in an unfamiliar environment.

Certain age groups of older owners were significantly more likely to report their rabbits to be unresponsive to an approach from behind and less likely to report their rabbits to respond to a treat being prepared than were 18–24 year olds (Table 5). No other rabbit or owner signalment factors reached statistical significance in predicting the rabbit behaviour tested.

## Discussion

We aimed to describe a range of ear conditions reported to affect UK pet rabbits, ear conformational risk factors for ear problems, how ear problems affected rabbit QoL, and which owner-reported behaviours were predictive of ear problems. The results broadly indicate that approximately a quarter of pet rabbits in this sample were reportedly affected by ear conditions that are painful and/or impair their hearing, and that lop-eared and half-lop rabbits are more prone to these than erect-eared rabbits are. Owners perceived rabbits with ear pain responses to have worse QoL than rabbits who did not show ear pain, but they did not perceive this effect on QoL in hearing impaired rabbits or those who had had ear problems indicated by a vet. The behaviours most indicative of vet indicated ear problems, ear pain, and hearing impairment were lack of responsiveness to relevant sounds, and a reduction in the frequency of binkying. Each set of results will be discussed in more detail.

### Description of ear disease and convergent validity

In this sample, more than a quarter of rabbits (28.5%) had had at least one ear problem reported. Our four measures of rabbits having ear conditions showed convergent validity, with statistically significant pairwise associations between veterinary indication of ear disease, owner reported ear problems affecting QoL, ear pain responses and hearing impairment. The lifetime prevalence of vet indicated ear problems for rabbits with a median (IQR) age of 4 (2–6) years was 21.2%. Moreover, 13.6% of rabbits were reported to have an ear problem that affected their QoL at the time of the survey, 7.1% had ear pain response, and 15.8 had owner reported hearing impairment or deafness (Table 2). The prevalences reported here are broadly consistent with, but slightly lower than, the 22–32% prevalences found in the studies that used post-mortem or retrospective CT scan analysis to identify otitis media and interna [13–15]; indeed 21% of the rabbits in the current survey had received a veterinary indication of otitis media or otitis interna, specifically, at some point in their lives. In contrast, the prevalences in this survey are considerably higher than the 1–3.5% prevalences estimated from veterinary clinical records [4, 5].

The prevalences here do not represent the whole UK pet rabbit population, because the sample population was self-selected and thus not a random sample, and the owner reports cannot be verified. The true prevalence could therefore be higher or lower. We attempted to avoid over-sampling rabbits with ear problems by wording our introductory text to be as much about healthy ears and excellent hearing ability as about the opposite, and indeed most respondents reported their rabbits not to have any ear problems. However, an inflated prevalence is possible because, compared with owners of rabbits without ear problems, those whose rabbits were affected could have been more likely to participate in the questionnaire, e.g. if they were concerned about the problem and wanted to communicate this concern to researchers. This would lead to an over-estimate of the prevalence. The opposite–an underestimate–is also possible, especially given that otitis media and hearing loss are difficult to recognise in rabbits [6, 7], meaning that some respondents might erroneously have believed their rabbits to be unaffected. Indeed two participants in this survey reported their rabbits as not having any of our four measures of ear problems, despite them regularly scratching at their ears, which is indicative of ear problems [1]. As a further illustration, BAER testing showed that dog owners could identify very good hearing and bilateral deafness in their dogs, but had poorer sensitivity and specificity for unilateral or intermediate levels of hearing impairment [47]. For the current study, it is also worth noting the female bias in the owner population, as is typical of many questionnaire populations. The only gender effect that we found was that men were significantly more likely to report ear pain responses than women were, so perhaps with a more gender-balanced sample population, the reported prevalence of ear pain would have been greater than in the current study.

Either way, the proportion of rabbits with ear conditions here is of concern, because the results indicate that such conditions are probably considerably more prevalent than the 1% of rabbits having auditory conditions per year as indicated by veterinary case notes from UK first opinion practices [4]. Our findings suggest that ear conditions may be being underdiagnosed, because some conditions are difficult to recognise in rabbits [6, 7], and a quarter of the affected rabbits reported in this survey had not received a veterinary indication of their condition. This lack of diagnosis could have occurred at least partly if some owners did not bring their rabbit to the vet for ear examination, even despite noticing ear pain or hearing impairment, perhaps to avoid veterinary fees or perhaps because owners did not feel any treatment was possible or necessary. The results here therefore indicate a risk of rabbits suffering from a lack of treatment of their ear conditions.

Otitis, especially otitis media and otitis interna, was the most common group of diagnosed conditions, according to the owners in this survey. Otitis of any type is of direct welfare concern, as it is likely to be very painful, can cause hearing loss and (in the case of otitis interna) vestibular disturbance such that rabbits develop head tilts or disorientation [1]. Otitis can additionally be a recurrent problem. Excess cerumen was also common, and whilst cerumen itself is not concerning in normal quantities, excess cerumen can lead to otitis as a secondary condition. Also, the physical build up of cerumen can block the ear canal, acting as a physical barrier to sound and thus causing reversible hearing impairment [48]. Additionally, the cerumen build up can result in ear base swellings, which may cause discomfort or pain [1]. Coupled with excess cerumen, otitis media and interna can be difficult to treat medically, because the cerumen blocks topical ear drops from entering the ear, so surgery may be required in severe and recurrent cases.

### Lop-eared phenotype and other signalment as risk factors for reported ear problems

In this study, lop-eared rabbits were predisposed to ear conditions, which is consistent with veterinary opinion [1, 6, 16, 17] and with previous studies [14–16]. In the current study, approximately 30% of lop-eared rabbits had a veterinary indication of ear disease, compared with 11% of erect-eared rabbits. Lop-eared conformation increased the risk of owners reporting their rabbits as having a vet indicated ear problem, an ear problem that reduced their QoL, ear pain responses, and hearing impairment. This is concerning, but it is worth noting that not all lop-eared rabbits in this study were affected. Instead, our results suggest that up to 70% of lop-eared rabbits could be unaffected by these problems, or at least not affected to a degree that owners perceive. It is encouraging if some lop-eared rabbits have the lop conformation without associated health problems, because it would offer promise in terms of breeders being able to select for healthy rabbits of this ear conformation in future.

Rabbit ear conformation is not a true dichotomy, and our population included half-lop and rabbits with horizontal ears. It is worth noting that 32% of half-lops had a vet indicated ear problem, similar to the prevalence in full-lops. There were only 18 horizontal-eared rabbits in the study, so it is difficult to draw conclusions about them, but they seemed to have a relatively low prevalence of 7% with a vet indicated ear problem. Whilst the numbers of these rabbits with intermediate ear conformations were sometimes too few for statistical analysis, the results suggest that half-lop rabbits may be at increased risk of certain ear problems compared with erect-eared rabbits, to a similar extent as rabbits with fully lop ears (Table 2). In particular, half-lop rabbits were four times more likely to have an ear problem thought to impair their QoL than erect eared ones were (Table 3). This implies that having even one lop-ear may be as much of a welfare risk for rabbits as having two lop ears.

All four measures of ear problems also increased with age, but for vet indicated problems and ear pain responses, this age effect was only seen in lop-eared rabbits. The likelihood of hearing impairment increases with age in some species, e.g. humans [49] and rats [50], but this has not previously been reported for rabbits. Our measure of vet indicated problems was a lifetime prevalence, so it will also have increased the longer that rabbits had been alive for, but it is notable that this increase was only seen in lop-eared rabbits, with a consistently low prevalence across all ages of erect-eared rabbits. Also, our results are consistent with the finding that otitis media as identified post-mortem was more prevalent in adult (32%) than juvenile rabbits (4%) [13]. Ear pain responses, which could signify otitis, increased with age, again only in lops. Ear mites were rarely reported in the current study, and too rare for analysis, but were found to be more common in juvenile rabbits than adults in a retrospective clinical study [5].

The increased risk of ear problems with age presents a practical challenge for rabbit breeders wanting to select for healthy ears. Female rabbits, in small breeds, tend to be bred between the ages of 4 months and 3 years, with larger breeds starting later around 10–12 months [51]. Males of certain breeds reach sexual maturity at 1.5 months and can sire litters for many years, so most litters may be born before the parents yet develop ear conditions. Early signs of ear disease predisposition, such as excess cerumen or stenotic ear canals, should be assessed when deciding whether to breed from a rabbit. Additionally, the ear health of the previous generation of rabbits should be taken into account.

### Reported effects of ear problems on QoL

Over two thirds (69.4%) of respondents who reported an ear problem in their rabbits felt it worsened the rabbits’ QoL. Additionally, rabbits with ear pain responses had significantly worse QoL scores than those without, suggesting that many owners recognise this as indicating suffering. It would be worth conducting objective welfare assessments of rabbits with ear conditions, because owner-reported QoL ratings are subjective. Even so, the results suggest that, regardless of rabbit ear conformation, ear diseases in rabbits should be regarded as a welfare issue and veterinary treatment should be sought.

An unexpected finding, which is at odds with the other results was that owners were slightly but statistically significantly more likely to describe their rabbit as ‘happy’ if it had a vet indicated ear problem than if it did not. This could be a Type I error (falsely significant), especially because almost all respondents selected ‘happy’ to describe their rabbit. Alternatively, it could mean that rabbits whose owners took them to a vet when they had an ear problem are perceived as ‘happier’ than other rabbits. This effect could occur directly through effective veterinary treatment of ear or other problems, or indirectly through some other features associated with having an owner who takes their rabbit to the vet, e.g. having a more attentive owner.

Another slightly unexpected finding was that hearing impairment and vet indication of ear problems were not significantly associated with worse QoL as perceived by owners. As above, it could be that rabbits who have been seen by a vet for an ear problem have had that condition resolved and are subsequently reported to have good QoL. It is possible that ear problems only worsen QoL if accompanied by pain, and that rabbits cope well with hearing impairment. Alternatively, it could be the case that hearing impairment, or a history of ear disease without obvious pain responses, do worsen rabbit QoL, but that the signs are too subtle for owners to consider them as clinically significant, e.g. rabbits being inactive, needing to be more visually vigilant, being more frequently startled, and missing positive sound cues that predict food or other rewards. The behaviours reported to be associated with both vet indicated ear problems and hearing impairment, discussed in the next section, suggest that the latter may be likely in many cases.

### Behavioural signs of ear problems

Owners of rabbits with vet indicated ear problems, ear pain responses, or hearing impairment were reported to be significantly less likely to respond to the sounds of treats being prepared, and significantly more likely to only rarely binky, compared with unaffected rabbits. This suggests that these ear problems blunt the rabbits’ abilities to experience positive welfare. Affected rabbits miss out on the pleasurable anticipation of a treat, and if socially housed, may be at a competitive disadvantage compared with conspecifics who can hear the treat being prepared and approach it earlier. Binkying is considered a play behaviour, so the reduced binkying frequency both reflects reduced welfare, because poor welfare reduces animals’ motivations to play, and causes poor welfare because play is pleasurable in itself [46]. Play is also a ‘low resilience’ behaviour, being energetic and yet relatively non-essential to survival, and such behaviours are often early indicators of poor welfare, because stressed or diseased animals conserve their energy to prioritise the essential behaviours [44]. Additionally, if the ear problems cause a depression-like state, then lack of responsiveness to a treat and lack of binkying could indicate anhedonia in the rabbits [52].

In addition to this, rabbits reported to have vet indicated ear problems or varying degrees of hearing impairment were also significantly less likely to respond to loud or threatening sounds, and to the sound of the owner approaching from behind. This could make them more vulnerable to attack or injury, and more likely to be startled by the sudden visual appearance of their owner, potential predators and other stimuli. Some rabbits may be able to habituate to this, but it might alternatively cause other rabbits to be almost constantly vigilant and potentially anxious, similar to the anxiety reported in hearing impaired humans [37]. Increased reports of these non-responsive behaviours in rabbits who have had vet indicated ear problems may be a result of more vigilant owners (due to knowledge of their rabbits ear problems), with owners of undiagnosed rabbits not noticing these potentially subtle signs. Similarly vigilant owners could be more attentive to their animal’s needs, thus causing the rabbit to startle less frequently or respond more subtly to acoustic cues.

These results offer insights into how ear problems affect rabbit behaviour and welfare beyond specific ear-directed behaviour reported in the literature, such as scratching of the ears or head shaking [1]. They also suggest that owners and vets should be vigilant of rabbits having reduced responsiveness to sounds, especially those predictive positive events, and having reduced binkying frequency as indicators of potential hearing impairment and other chronic diseases. As suggested in veterinary texts, ear-directed behaviour can additionally be used to suggest that rabbits have an ear problem specifically. Indeed, even of the rabbits whose owners reported their rabbits to have an ear problem that worsened QoL, to show ear pain responses, or to have impaired hearing, almost one quarter had received no veterinary indication of ear disease, perhaps because they did not take their rabbit to the vet for these problems. It should be noted that all the results in the current study are owner reported, so they require verification using systematic behaviour analysis.

## Conclusions

Ear problems in rabbits are associated with owner-reported reductions in QoL, reduced responsiveness to sounds, and reduced binkying behaviour. They may be painful and cause hearing impairments. Lop- and half-lop-eared rabbits are more at risk than erect-eared rabbits are, as are older rabbits. Breeders wanting to select for good ear health should avoid breeding from rabbits with early signs of ear disease or with a family history of it. Ear disease can be difficult to recognise in rabbits, so it may be under-diagnosed and under-treated, but increased recognition of it is needed because of its associated harms to rabbit welfare.

## Supporting information

S1 File Full questionnaire. (PDF)

S2 File Supplementary methods: Response collapsing for binary variables. (DOCX)

S3 File Supplementary results: Responses to looking inside the rabbit’s ear. (DOCX)
