# Supplementary material for: Ear health and quality of life in pet rabbits of differing ear conformations: A UK survey of owner-reported signalment risk factors and effects on rabbit welfare and behaviour
Source: PLoS One. 2023 Jul 19;18(7):e0285372. doi: 10.1371/journal.pone.0285372 (PMC10355490; doi:10.1371/journal.pone.0285372)
Supplement: S1 File — (PDF) [file pone.0285372.s001.pdf]

Rabbit Ear Health and Hearing Impairments Questionnaire

Through this Royal Veterinary College research, we hope to understand how both good and bad ear health and hearing impairments affect rabbit welfare and overall quality of life. To complete this research, we require owners of rabbits, whether the rabbit has excellent hearing or seems to have sore ears or difficulty hearing, to complete this questionnaire.

This anonymous questionnaire is open to anyone who is over the age of 18 and currently owns a rabbit. This questionnaire will take approximately 10 minutes to complete and includes 29 questions. The questionnaire is made up of 4 parts: 'About You', 'About Your Rabbit', 'Your Rabbit's Quality of Life' and 'Your Rabbit's Hearing'.

This study is being conducted by a Royal Veterinary College student, as part of a final year Veterinary Medicine research project. This questionnaire is completely anonymous, and you may withdraw from the study at any time by leaving the questionnaire pages. You must be 18 years of age or over.

This study is intended for educational purposes only, and not for commercial purposes. The results may be published in a peer-reviewed journal and presented at relevant professional conferences. Your data will be held in accordance with the Data Protection Act 1998 and the General Data Protection Regulation 2018.

The questionnaire is expected to close on the 31st January 2022.

Principal Researcher: Melissa Keeler, Veterinary Medicine Student at the Royal Veterinary College
Email: mkeeler6@rvc.ac.uk
Project Supervisor: Charlotte Burn, Associate Professor in Animal Welfare and Behaviour Science at the Royal Veterinary College
Email: cburn@rvc.ac.uk
Ethical approval for this project has been given by the Social Science Research Ethical Review Board at the Royal Veterinary College (Reference: URN SR2021-0167).

* 1. I confirm I would like to opt in to participate in this survey

- ◯ Yes (proceed to next page)
- ◯ No (exit questionnaire)

Rabbit Ear Health and Hearing Impairments Questionnaire

About You

This section is purely so that we can understand the general demographics of the survey participants.

2. What is your gender?

- Female
- Male
- Non-Binary
- Prefer not to say

3. What is your age?

- Under 18
- 18-24
- 25-34
- 35-44
- 45-54
- 55-64
- 65+

4. Which country do you live in?

- U.K.
- E.U. country (please state)
- U.S.A.
- Canada
- Australia
- Other (please state)

Other (please specify)

5. Do you work with rabbits?

- No
- Yes, I am a veterinary professional
- Yes, I am a veterinary student (e.g. veterinary medicine, veterinary nurse etc)
- Yes, I work with rabbits in another context (e.g. rescue, charity, breeder, scientist etc) - please give details
- No, but I used to - please give details
- Other - please give details

Other (please specify)

Rabbit Ear Health and Hearing Impairments Questionnaire

About Your Rabbit

* 6. How many rabbits do you currently own?

- 1
- 2
- 3 or more
- None (Please explain)

Other (please specify)

If you own more than one rabbit, please answer this questionnaire for only one rabbit. If you are having difficulty choosing which rabbit, pick the rabbit whose name comes earliest in the alphabet.

7. What sex is the rabbit you are answering this questionnaire about?
(If you have more than one rabbit and are having difficulty choosing which rabbit, pick the rabbit whose name comes earliest in the alphabet.)

- Male entire
- Male neutered
- Female entire
- Female neutered
- Unsure

8. How old is your rabbit?

- ◯ Less than 1 year
- ◯ 1
- ◯ 2
- ◯ 3
- ◯ 4
- ◯ 5
- ◯ 6
- ◯ 7
- ◯ 8
- ◯ 9
- ◯ 10+
- ◯ Unsure

9. What breed is your rabbit?

- ◯ Dwarf Lop/Mini Lop
- ◯ Netherland Dwarf
- ◯ Sussex
- ◯ Himalayan
- ◯ New Zealand White
- ◯ Californian
- ◯ Dutch
- ◯ Long-haired breed, e.g. Angora
- ◯ French lop
- ◯ Mini Rex
- ◯ Continental Giant
- ◯ Standard Rex
- ◯ Lionhead
- ◯ English lop
- ◯ Crossbreed
- ◯ Unknown

Other (please specify)

* 10. Is your rabbit lop-eared or erect-eared?

- Lop-eared
- Erect-eared
- One ear up, one down
- Intermediate, i.e. approximately horizontal
- Unsure

Royal Veterinary College
University of London

Rabbit Ear Health and Hearing Impairments Questionnaire

Your Rabbit's Quality of Life

* 11. How would you rate your rabbit’s quality of life (considering health, stress levels, ability to express natural behaviours, companionship etc.)?

- Very good
- Good
- Moderate
- Bad
- Very bad
- Unsure

Other (please specify)

* 12. Does your rabbit have any health problems that you believe affects their quality of life?

- Yes, an ear or hearing problem (please specify)
- Yes, another health problem (please specify)
- Yes, both an ear problem and another health problem (please specify)
- No - my rabbit does have an ear problem, but it does not seem to affect their quality of life
- No
- Unsure

Other (please specify)

13. Does your rabbit enjoy being stroked around their face/ears?

- Yes
- Sometimes
- Not particularly
- No
- Unsure

14. Does your rabbit play with e.g. toys, other rabbits, or familiar people?

- Yes
- Sometimes
- Not particularly
- No
- Unsure

15. How frequently does your rabbit binky (jumping with all four paws off the ground, often seen during play)?

- Often
- Sometimes
- Rarely
- Never
- Unsure

16. If you have recently needed to put your rabbit in an unfamiliar, new place (e.g. a holiday pen, a new play area, or an unfamiliar room), what best describes how he/she reacted within the first hour or so?
If you do not know, please select 'Unsure'. Do NOT put your rabbit in an unfamiliar place for the purpose of this survey, because this would cause unnecessary stress.

- Rabbit would actively explore, hopping, rearing and sniffing
- Rabbit would slowly and cautiously explore, sniffing, and moving a little at a time
- Rabbit would freeze and try to hide
- Rabbit would mostly sit still with its back in a corner, facing the unfamiliar space
- Unsure

Other (please specify)

17. From your knowledge of your rabbit, would you say that overall, they are (please tick all that apply):

- [ ] Happy
- [ ] Relaxed
- [ ] Nervous/fearful
- [ ] Worried
- [ ] Stressed
- [ ] Angry
- [ ] Curious
- [ ] Unsure
- [ ] Other (please specify)

Rabbit Ear Health and Hearing Impairments Questionnaire

Your Rabbit's Hearing

* 18. How would you describe your rabbit's hearing ability?

- ◯ Very good – responds to the slightest sound
- ◯ Good – responds to most relevant sounds
- ◯ Impaired – responds only to certain loud sounds
- ◯ Deaf – does not respond to sounds
- ◯ Unsure

19. What makes you think this? Tick all that apply to your rabbit

- ☐ Rabbit generally moves her/his ears most of the time, as if scanning the environment (not applicable to some lop eared rabbits)
- ☐ Rabbit's ears move little if at all despite the rabbit being able to move them (not applicable to some lop eared rabbits)
- ☐ Rabbit pricks her/his ears at relevant sounds (not applicable to some lop eared rabbits)
- ☐ Rabbit turns his/her head towards relevant sounds
- ☐ Rabbit turns his/her head and looks around frequently whether or not there are obvious sounds
- ☐ Rabbit sometimes gets woken up by sounds
- ☐ Rabbit is generally unresponsive to sounds
- ☐ Unsure

Other (please specify)

20. What best describes what your rabbit does when you approach from a direction where (s)he cannot see you, e.g. if you approach from behind?

- Rabbit turns its head and body towards me when I approach from a distance
- Rabbit turns ears towards me, and freezes or runs and hides as I approach from a distance
- Rabbit initially does not react and then often seems startled, jumping when I approach and touch her/him from behind
- Rabbit does not respond to my approach unless (s)he is facing me
- Unsure
- Other (please specify)

21. What does your rabbit do when you prepare a treat within earshot of him/her (e.g. open a rabbit food packet)?

- Rabbit turns its head and body towards sounds associated with the treat
- Rabbit does not respond to sounds associated with food, unless (s)he can see or smell the treat
- Rabbit is not interested in treats, even if (s)he can see or smell them
- Rabbit runs away from the sound
- Unsure

Other (please specify)

22. What does your rabbit do when there are loud or threatening sounds?

- Rabbit freezes (e.g. stops eating and stays very still)
- Rabbit runs and hides
- Rabbit turns towards the sound source
- Rabbit does not seem to notice
- Unsure

Other (please specify)

23. Is there anything else your rabbit does that leads you to believe it can OR cannot hear well?

Rabbit Ear Health and Hearing Impairments Questionnaire

Your Rabbit's Health

24. How frequently does your rabbit require ear examination or ear cleaning by a vet?

- ○ Every month
- ○ Every 6 months
- ○ Every year
- ○ Never
- ○ Unsure

25. If you gently look into your rabbit's ears, what do you sometimes see? (please tick all that apply)

- ☐ A small amount of ear wax (yellow)
- ☐ The skin in the ear seems somewhat red
- ☐ The hole of the ear canal is visible
- ☐ The hole of the ear canal is not visible
- ☐ Brown crusting
- ☐ One or more lumps
- ☐ One or more small bumps or spots
- ☐ Rabbit flinches and pulls away
- ☐ Hair/fur
- ☐ Scratches or cuts
- ☐ Pus
- ☐ Unpleasant smell
- ☐ Clean, healthy skin with no obvious redness
- ☐ Unsure

Other (please specify)

26. What does your rabbit do if you gently try to look into their ears? (tick all that apply)
Please do not do this for the purpose of this survey, as it could cause unnecessary stress, so we are asking about previous times you have done this.

- [ ] Increase in respiration (fast breathing)
- [ ] Lying down, with a relaxed posture (and ears upright if not a lop-eared rabbit)
- [ ] Freezing, hunched up (and attempting to flatten its own ears against the body if not a lop-eared rabbit)
- [ ] Reluctance to move
- [ ] Thumping its hind feet
- [ ] Nose twitching
- [ ] Sudden aggression (e.g. attempting to bite)
- [ ] Persistently squinting the eyes
- [ ] Being excessively jumpy and watchful (bulging eyes)
- [ ] Struggling or moving away
- [ ] Nothing noticeable and seems relaxed
- [ ] Unsure (e.g. I have not tried this or cannot remember)

Other (please specify)

27. Do you think your rabbit behaves as if it is painful when you try to look into their ears?

- ◯ Yes, definitely
- ◯ Yes, somewhat
- ◯ No
- ◯ Unsure

28. Does your rabbit have a head tilt (appearing to have her/his head tipped to one side all the time)?

- ◯ Yes, definitely
- ◯ Yes, somewhat
- ◯ My rabbit has had a head tilt in the past, but no longer does
- ◯ No
- ◯ Unsure

* 29. Has a vet ever diagnosed your rabbit as having any ear problem(s)?

- ◯ Yes
- ◯ The vet mentioned it, but did not formally diagnose it
- ◯ No
- ◯ Unsure

30. (If yes or a vet has mentioned it), what did your rabbit suffer from?

- [ ] Ear abscess
- [ ] Inflammation or infection of the outer ear (Otitis externa)
- [ ] Middle ear infection (Otitis media)
- [ ] Inner ear infection, vestibular disease or labyrinthitis (Otitis interna)
- [ ] Unspecified ear infection (Otitis)
- [ ] Ear mites
- [ ] Excessive ear wax
- [ ] Cut or other injury to the outer ear
- [ ] Allergy affecting the ear
- [ ] Hearing loss
- [ ] Unsure
- [ ] Not applicable

Other (please specify)

31. How old was your rabbit when they were first diagnosed with any of the problems related to their ears?

- ○ Less than 1 year
- ○ 1
- ○ 2
- ○ 3
- ○ 4
- ○ 5
- ○ 6
- ○ 7
- ○ 8
- ○ 9
- ○ 10+
- ○ Unsure
- ○ My rabbit has never been diagnosed with any problems related to their ears

Thank you for completing the questionnaire!

If you have any questions about the survey, you can contact the research supervisor, Dr Burn: cburn@rvc.ac.uk; or the research student, Ms Keeler: mkeeler6@rvc.ac.uk.

If you are concerned about your rabbit's ears or hearing, do take her/him to a veterinarian to be checked. You can also find information on the following pages:
https://www.pdsa.org.uk/taking-care-of-your-pet/looking-after-your-pet/rabbits/rabbit-body-language
https://www.rspca.org.uk/adviceandwelfare/pets/rabbits/health
https://rabbitwelfare.co.uk/rabbit-health/
https://www.thehealthypetclub.co.uk/ear-problems-rabbits/
https://www.unusualpetvets.com.au/lop-eared-rabbits-and-ear-disease/
https://rabbit.org/head-tilt-causes-and-treatment/

32. If you have any further comments, please do let us know.
