# Supplementary material for: Ear health and quality of life in pet rabbits of differing ear conformations: A UK survey of owner-reported signalment risk factors and effects on rabbit welfare and behaviour
Source: PLoS One. 2023 Jul 19;18(7):e0285372. doi: 10.1371/journal.pone.0285372 (PMC10355490; doi:10.1371/journal.pone.0285372)
Supplement: S2 File — (DOCX) [file pone.0285372.s002.docx]

# S2 Supplementary Methods: Response collapsing for binary variables

For the question ‘Has a vet ever diagnosed your rabbit as having any ear problem(s)?’, categories of ‘Yes’ and ‘No’ were formed from the responses of ‘Yes’ and ‘No’ respectively. To assess in which binary category ‘The vet mentioned it, but did not formally diagnose it’ should be placed, the distribution of reported pain when having their ears looked in across each vet category was analysed using a Kruskal-Wallis test and subsequent posthoc Dunn’s pairwise tests on each pair, adjusted using the Bonferroni correction. The distribution of pain was different between the groups (χ2(2) = 101.49, *P* = < 0.001), with pairwise comparisons indicating a significant difference between ‘no’ and ‘mention’ (*P* = < 0.001), and ‘no’ and ‘yes’ (*P* = < 0.001). There was no significant difference between ‘mention’ and ‘yes’ (*P* = 0.687), Therefore, responses of ‘The vet mentioned it, but did not formally diagnose it’ were included in the ‘Yes’ category. Further collapsing schemes for binary variables are presented in Table S1.

**Table S1**. Category collapsing scheme for question responses when a binary variable was required for analysis. *Asterisks represent questions in which free text answers were assigned to categories manually if they clearly indicated responsiveness (or not) in relation to the question.

| **Question** | **Original responses** | **Collapsed category** |
| --- | --- | --- |
| Has a vet ever diagnosed your rabbit as having any ear problem(s)? (see text for details). | Yes  The vet mentioned it, but did not formally diagnose it | Yes |
|  | No | No |
| Do you think your rabbit behaves as if it is painful when you try to look into their ears? | Yes, definitely  Yes, somewhat | Yes |
|  | No | No |
| How would you describe your rabbit’s hearing ability? | Very good – responds to the slightest sound  Good – responds to most relevant sounds | Good |
|  | Impaired – responds only to certain loud sounds  Deaf – does not respond to sounds | Impaired |
| Does your rabbit have any health problems that you believe affects their QoL? | Yes, an ear or hearing problem  Yes, both an ear problem and another health problem | Yes |
|  | Yes, another health problem  No - my rabbit does have an ear problem, but it does not seem to affect their QoL  No ear or other problems  No | No |
| What best describes what your rabbit does when you approach from a direction where (s)he cannot see you, e.g. if you approach from behind?* | Rabbit turns its head and body towards me when I approach from a distance  Rabbit turns ears towards me, and freezes or runs and hides as I approach from a distance | Responsive |
|  | Rabbit initially does not react and then often seems startled, jumping when I approach and touch her/him from behind  Rabbit does not respond to my approach unless (s)he is facing me | Unresponsive |
| What does your rabbit do when you prepare a treat within earshot of him/her (e.g. open a rabbit food packet)?* | Rabbit turns its head and body towards sounds associated with the treat | Responsive |
|  | Rabbit does not respond to sounds associated with food, unless (s)he can see or smell the treat | Unresponsive |
|  | Rabbit is not interested in treats, even if (s)he can see or smell them | Unsure |
| What does your rabbit do when there are loud or threatening sounds?* | Rabbit freezes (e.g. stops eating and stays very still)  Rabbit runs and hides  Rabbit turns towards the sound source | Responsive |
|  | Rabbit does not seem to notice | Unresponsive |
| How frequently does your rabbit binky (jumping with all four paws off the ground, sometimes seen during play)? | Often  Sometimes | Sometimes |
|  | Rarely  Never | Rarely |
| If you have recently needed to put your rabbit in an unfamiliar, new place (e.g. a holiday pen, a new play area, or an unfamiliar room), what best describes how he/she reacted within the first hour or so? If you do not know, please select 'Unsure'. Do NOT put your rabbit in an unfamiliar place for the purpose of this survey, because this would cause unnecessary stress. | Rabbit would freeze and try to hide  Rabbit would mostly sit still with its back in a corner, facing the unfamiliar space | Cautious |
|  | Rabbit would slowly and cautiously explore, sniffing, and moving a little at a time  Rabbit would actively explore, hopping, rearing and sniffing | Explorative |
