# Supplementary material for: Ear health and quality of life in pet rabbits of differing ear conformations: A UK survey of owner-reported signalment risk factors and effects on rabbit welfare and behaviour
Source: PLoS One. 2023 Jul 19;18(7):e0285372. doi: 10.1371/journal.pone.0285372 (PMC10355490; doi:10.1371/journal.pone.0285372)
Supplement: S3 File — (DOCX) [file pone.0285372.s003.docx]

# S3 Supplementary Results: Responses to looking inside the rabbits ear

**Table S2**. Number of reports for responses to ‘gently looking in the ear’ for each ear conformation and by veterinary indication. Multiple specific responses could be selected for the same rabbit. Brackets indicate percentage of responses for the question for each ear conformation or vet indication category. *Unfortunately, the complementary category of ‘a large amount of ear wax (yellow)’ was missing from the final version of the questionnaire.

| **Responses** | **Ear conformation** |  |  |  | **Vet indication** |  | **Total (n)** |
| --- | --- | --- | --- | --- | --- | --- | --- |
|  | **Erect (n)** | **Lop (n)** | **Asymmetrical (n)** | **Horizontal (n)** | **Yes (n)** | **No (n)** |  |
| Total respondents providing at least one answer to question | 202 | 231 | 28 | 15 | 102 | 376 | 478 |
| Clean, healthy skin with no obvious redness | 161 (79.7%) | 161 (69.7%) | 20 (71.4%) | 12 (80.0%) | 48 (47.1%) | 306 (81.4%) | 354 (74.1%) |
| The hole of the ear canal is visible | 65 (32.2%) | 60 (26%) | 7 (25.0%) | 9 (60.0%) | 22 (20.6%) | 118 (31.4%) | 139 (29.1%) |
| Hair/fur | 31 (15.3%) | 27 (11.7%) | 2 (7.1%) | 6 (40%) | 10 (9.8%) | 56 (14.9%) | 66 (13.8%) |
| A small amount of ear wax (yellow)* | 10 (5%) | 30 (13%) | 3 (10.7%) | 0 (0%) | 30 (29.4%) | 13 (3.5%) | 43 (9%) |
| Rabbit flinches and pulls away | 9 (4.5%) | 11 (4.8%) | 2 (7.1%) | 1 (6.7%) | 11 (10.8%) | 12 (3.2%) | 23 (4.8%) |
| One or more small bumps or spots | 6 (3.0%) | 14 (6.1%) | 2 (7.1%) | 0 (0%) | 8 (7.8%) | 14 (3.7%) | 22 (4.6%) |
| The hole of the ear canal is not visible | 4 (2.0%) | 13 (5.6%) | 2 (7.1%) | 1 (6.7%) | 11 (10.8%) | 9 (2.4%) | 20 (4.2%) |
| Scratches or cuts | 6 (3%) | 6 (2.6%) | 1 (3.6%) | 0 (0%) | 6 (6.9%) | 7 (1.9%) | 13 (2.7%) |
| The skin in the ear seems somewhat red | 6 (3%) | 4 (1.7%) | 1 (3.6%) | 0 (0%) | 10 (9.8%) | 2 (0.5%) | 12 (2.5%) |
| Pus | 0 (0%) | 9 (3.9%) | 1 (3.6%) | 0 (0%) | 10 (9.8%) | 0 (0%) | 10 (2.1%) |
| One or more lumps | 1 (0.5%) | 7 (3.0%) | 0 (0%) | 0 (0%) | 8 (7.8%) | 1 (0.3%) | 8 (1.7%) |
| Brown crusting | 3 (1.5%) | 3 (1.3%) | 1 (3.6%) | 0 (0%) | 6 (5.9%) | 1 (0.3%) | 7 (1.5%) |
| Unpleasant smell | 0 (0%) | 6 (2.6%) | 1 (3.6%) | 0 (0%) | 7 (6.9%) | 0 (0%) | 7 (1.5%) |
